# Allergic airway inflammation induces the migration of dendritic cells into airway sensory ganglia

**DOI:** 10.1186/1465-9921-15-73

**Published:** 2014-06-30

**Authors:** Duc Dung Le, Sabine Rochlitzer, Axel Fischer, Sebastian Heck, Thomas Tschernig, Martina Sester, Robert Bals, Tobias Welte, Armin Braun, Quoc Thai Dinh

**Affiliations:** 1 Department of Experimental Pneumology and Allergology, Saarland University Faculty of Medicine, Kirrberger Strasse, Geb. 61.4, Homburg, Germany; 2 Department of Airway Immunology, Fraunhofer Institute for Toxicology and Experimental Medicine, German Centre for Lung Research (DZL), Hannover, Germany; 3 Institute of Occupational Medicine, Charité - Universitätsmedizin Berlin, Free University and Humboldt University, Berlin, Germany; 4 Institute of Anatomy and Cell Biology, Saarland University Faculty of Medicine, Homburg, Germany; 5 Department of Transplant and Infection Immunology, Saarland University, Homburg, Germany; 6 Department of Internal Medicine V, Pneumology, Allergology and Respiratory Critical Care Medicine, Saarland University Faculty of Medicine, Kirrberger Strasse, Geb. 61.4, Homburg, Germany; 7 Department of Pneumology, Hannover Medical School, German Centre for Lung Research (DZL), Hannover, Germany

**Keywords:** House dust mite mouse model, Dendritic cells, Allergic airway inflammation, Sensory airway nerves, Neuroimmune interaction, CGRP

## Abstract

**Background:**

A neuroimmune crosstalk between dendritic cells (DCs) and airway nerves in the lung has recently been reported. However, the presence of DCs in airway sensory ganglia under normal and allergic conditions has not been explored so far. Therefore, this study aims to investigate the localisation, distribution and proliferation of DCs in airway sensory ganglia under allergic airway inflammation.

**Methods:**

Using the house dust mite (HDM) model for allergic airway inflammation BALB/c mice were exposed to HDM extract intranasally (25 μg/50 μl) for 5 consecutive days a week over 7 weeks. With the help of the immunohistochemistry, vagal jugular-nodose ganglia complex (JNC) sections were analysed regarding their expression of DC-markers (MHC II, CD11c, CD103), the neuronal marker PGP 9.5 and the neuropeptide calcitonin gene-related peptide (CGRP) and glutamine synthetase (GS) as a marker for satellite glia cells (SGCs). To address the original source of DCs in sensory ganglia, a proliferation experiment was also carried in this study.

**Results:**

Immune cells with characteristic DC-phenotype were found to be closely located to SGCs and vagal sensory neurons under physiological conditions. The percentage of DCs in relation to neurons was significantly increased by allergic airway inflammation in comparison to the controls (HDM 51.38 ± 2.38% vs. control 28.16 ± 2.86%, p < 0.001). The present study also demonstrated that DCs were shown to proliferate in jugular-nodose ganglia, however, the proliferation rate of DCs is not significantly changed in the two treated animal groups (proliferating DCs/ total DCs: HDM 0.89 ± 0.38%, vs. control 1.19 ± 0.54%, p = 0.68). Also, increased number of CGRP-positive neurons was found in JNC after allergic sensitisation and challenge (HDM 31.16 ± 5.41% vs. control 7.16 ± 1.53%, p < 0.001).

**Conclusion:**

The present findings suggest that DCs may migrate from outside into the ganglia to interact with sensory neurons enhancing or protecting the allergic airway inflammation. The increase of DCs as well as CGRP-positive neurons in airway ganglia by allergic airway inflammation indicate that intraganglionic DCs and neurons expressing CGRP may contribute to the pathogenesis of bronchial asthma. To understand this neuroimmune interaction in allergic airway inflammation further functional experiments should be carried out in future studies.

## Introduction

Allergic bronchial asthma is a chronic inflammatory respiratory disease characterised by airway obstruction, bronchial hyperreactivity and airway inflammation with the recruitment of a variety of immune cells including dendritic cells (DCs) [1-3]. DCs are phagocytic cells that are localised in many organs like in the skin, in the mucosa of the intestines, the upper airways, the lungs and the brain [2-5].

In the allergic sensitisation phase, DCs play a key role as professional antigen presenting cells in the allergic airway inflammation [3,4,6]. They capture the antigen, process and subsequently present it on the MHC class II molecules (MHC II) to naïve T lymphocytes in local lymph nodes leading to cascades of the Th2-immune allergic inflammatory processes [4,7,8]. Recently, the maturation and differentiation of DCs have been described to be modulated by many cytokines of immune cells as well as neuropeptides such as calcitonin gene-related peptide (CGRP) [9-11].

CGRP consists of 37 amino acids [12] and is biosynthesised and released from sensory neurons innervating the airways in response to different stimuli including allergic airway inflammation [12-14]. CGRP released from airway nerve fibres has the capacity to act as chemoattractant factor for different immune cells such as CD4^+^ T-lymphocytes, CD8^+^ T-lymphocytes, eosinophils and DCs and to induce the proliferation of airway epithelial cells [9,15-19]. On the other hand, DCs have the capacity to release neurotrophins, which can activate neurons leading to the production of neuropeptides causing neurogenic airway inflammation [20,21]. Previously, DCs were found to be frequently associated anatomically with CGRP-containing sensory nerve fibres of the airways and skin [22,23].

Peripheral airway sensory nerve fibres are known to be derived from the neuronal cell bodies which are located in jugular-nodose ganglia complex (JNC) and able to produce, store and release neuropeptides such as tachykinins and CGRP to cause neurogenic inflammation [24-26]. However, DCs in airway sensory ganglia have not been explored under normal and allergic airway conditions so far.

The present study, therefore, aimed to investigate the localisation, distribution and proliferation of DCs and CGRP immunoreactive (IR)-neurons in vagal sensory jugular-nodose ganglia under allergic airway inflammation by using a chronic house dust mite (HDM) mouse model.

## Materials and methods

### Animals

Female wild-type BALB/c-mice (6–8 weeks old) were purchased from Charles River. The animals were held in regular 12 h dark/light cycles at a temperature of 22°C and received laboratory food and tap water *ad libitum*. The animals were acclimatised for at least 2 weeks prior to the study. All animal experiments were performed in strict concordance with the German animal protection law and approved by the appropriate governmental authority (No. 10/0207 and 14/2013).

### HDM-mouse model

**Figure 1:**
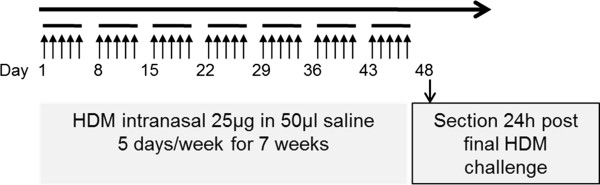
**Allergic sensitisation and challenge protocol of chronic HDM mouse model.** The animals were systemically sensitised by intranasal application of house dust mite 5 days a week for 7 weeks. All analyses were carried out 24 hours after the final challenge.

To induce chronic allergic airway inflammation, BALB/c mice (n = 10) were exposed consecutively 5 days a week within a total period of 7 weeks by intranasal instillation of HDM extract (Greer Inc., Cat. No XPB82D3A2.5) with a dose of 25 μg protein in 50 μl saline. A second group of animals (n = 10) served as control and was treated intranasal with 50 μl saline. Analyses were performed 24 h after the last allergen challenge (Figure 1) [27,28].

### In vivo proliferation study with EdU (5-ethynyl-2’-deoxyuridine)

EdU is a nucleoside analog of thymidine and is incorporated into DNA during active DNA synthesis. Controls and HDM-treated animals received an i.p. injection of 1 mg EdU (Invitrogen) in a volume of 200 μl 24 h before sacrifice.

### Bronchoalveolar lavage fluid (BALF)

Bronchoalveolar lavage was performed by instillation of 0.8 ml ice-cold PBS twice as described before [10]. The BALF was centrifuged (320 × g, 10 min, 4°C) and the supernatants were removed. The cell pellets were resuspended in 0.5 ml PBS and counted automatically with a Casy® cell counter. Cytospots were prepared, stained according to Pappenheim and differential cell counts were evaluated.

### Lung histology

For the assessment of the airway inflammation, acetone fixed lung cryosections of allergen sensitised/challenged and control animals were stained with hematoxylin and eosin. Cryosections (10 μm) were prepared using a cryostat (CM1950; Leica Cryostat, Nussloch, Germany) and stained with hematoxylin and eosin (H&E) and Periodic Acid Schiff (PAS) according to standard protocols.

### Ganglion preparation

JNC ganglia were fixed in Zamboni solution (Morphisto – Evolutionsforschung und Anwendung GmbH, Frankfurt am Main, Germany) for six hours, rinsed overnight in 0.1 M phosphate buffered saline (PBS) and cryoprotected 24 h with 30% sucrose in 0.1 M PBS. All steps above were performed at 4°C. The ganglia were embedded in OCT and frozen in liquid nitrogen. Serial 8 μm sections of the ganglia were prepared using a cryostat (Leica CM 1900, Bensheim, Germany), placed on APES (3-aminopropyltriethoxysilane) coated glass slides, dried at room temperature for 30 min and then stored in the freezer at −80°C.

### Immunofluorescence staining

Every fourth section from serial sections of the JNC was dried at room temperature for 15 min and then rehydrated for 5 min in PBS. To reduce nonspecific antibody binding, the sections were incubated for 15 min at room temperature in 5% normal serum of the host species of the secondary antibody diluted in PBS. The sections were incubated with primary antibodies against PGP 9.5, MHC II, GS, CD11c, CD103, CD11b, F4/80, Iba1, GFAP, CGRP, or the appropriate isotype control antibodies (listed in Table 1) for 1 h at room temperature and then overnight at 4°C. After rinsing with 0.1 M PBS twice, the sections were incubated with secondary fluorescein conjugated antibodies (listed in Table 1) for 2 h at room temperature, for counterstaining the section then were incubated with 100 μl DAPI (0.5 μg/ml, Carl Roth, Germany) for 15 min at room temperature. Finally the sections were washed twice with 0.1 M PBS, once with double distilled water, mounted with fluorescent mounting medium Prolong Gold (Invitrogen) and covered with cover slips.

**Table 1:** List of antibodies used in this study

**Antibody**	**Source**	**Dilution**
***Primary purified antibodies***		
Rabbit polyclonal antibody against mouse, human PGP 9.5	Abcam, Cambridge, UK	1:200
Rat monoclonal antibody against mouse I-A/I-E	BioLegend, San Diego, USA	1:200
Goat polyclonal antibody against mouse CGRP	Acris, Herford, Germany	1:400
Armenian hamster monoclonal antibody against mouse CD11c	Abcam, Cambridge, UK	1:100
Armenian hamster monoclonal antibody against mouse CD103	eBioscience, San Diego, USA	1:100
Rat monoclonal antibody against mouse CD11b	eBioscience, San Diego, USA	1:100
Rat monoclonal antibody against mouse F4/80	eBioscience, San Diego, USA	1:100
Rabbit polyclonal antibody against mouse glutamine synthetase (GS)	Abcam, Cambridge, UK	1:500
Rabbit Rabbit polyclonal antibody against Iba1	Wako Chemicals, Japan	1:300
Goat polyclonal antibody against GFAP	Abcam, Cambridge, UK	1:200
***Isotype control purified antibodies***		
Rabbit IgG	Dianova, Hamburg, Germany	1:5000
Rat IgG 2b kappa	BioLegend, San Diego, USA	1:200
Goat IgG	R&D systems,	1:300
Armenian Hamster IgG	BioLegend, San Diego, USA	1:200
***Secondary antibodies***		
DyLight 488 conjugated Donkey Anti-Rabbit IgG antibody	Jackson ImmunoResearch, INC., Baltimore, USA	1:400
Cy3 conjugated Donkey Anti-Rat IgG antibody	Jackson ImmunoResearch, INC., Baltimore, USA	1:500
Cy3 conjugated Donkey Anti-Rabbit IgG antibody	Jackson ImmunoResearch, INC., Baltimore, USA	1:400
DyLight 488 conjugated Donkey Anti-Goat IgG antibody	Jackson ImmunoResearch, INC., Baltimore, USA	1:400
Dylight 649 conjugated Goat Anti-Armenian Hamster IgG antibody	Jackson ImmunoResearch, INC., Baltimore, USA	1:400
DyLight 649 conjugated Donkey Anti-Rabbit IgG antibody	Jackson ImmunoResearch, INC., Baltimore, USA	1:400
Cy3 Conjugated Donkey Anti-Goat IgG	Jackson ImmunoResearch, INC., Baltimore, USA	1:500
Cy5 conjugated Donkey Anti-Rat IgG antibody	Jackson ImmunoResearch, INC., Baltimore, USA	1:400

Quantitative analysis of MHC II IR-cells, CGRP ir-neurons and PGP 9.5 ir-neurons was visualised with epifluorescence microscopes (Axioskop 2 plus, Carl Zeiss and Olympus BX5), and cell counting was performed using the software ImageJ (National Institutes of Health). The evaluations of MHC II IR-cells and CGRP IR-neurons were normalised by the total neuron number and expressed as percentages.

Confocal images were acquired by LSM 510 META (Carl Zeiss, Jena, Germany). The Images were processed using Imaris 4.5.2 (Bitplane, Zurich, Switzerland).

### EdU staining and quantification of EdU-labelled cells in JNC

EdU staining was conducted using Click-iT™ Cell Reaction Buffer Kit and Alexa Fluor 594 azide (Invitrogen) according to the manufacture’s protocol. The JNC sections were rehydrated for 5 min in PBS and the blocked with 5% normal serum. The sections were incubated with 200 μl prepared Click-iT reaction cocktail for 30 min and then incubated with primary and secondary antibodies as described above. Primary antibodies included rat anti-mouse MHC class II (Biolegend) and rabbit anti-mouse PGP 9.5 (Abcam). Secondary detection was performed with donkey anti-rat Cy2 and donkey anti-rabbit DyLight 647 (Jackson ImmunoResearch) (Table 1). DAPI was used for counterstaining.

For the quantification, 16 sections of each mouse were analysed. Only the EdU-labelled and MHC II- and DAPI-positive cells were observed for quantification of proliferating cells. Evaluations of proliferating, MHC II positive cells with an EdU-labelled nucleus were normalised with the total MHC II positive cells and expressed as percentages.

### Statistical analysis

Data is given as mean ± SEM. Statistical significance between groups was analysed with unpaired t-test using GraphPad Prism 4.03. Results of P values < 0.05 were considered significant.

## Results

### HDM induces allergic airway inflammation

**Figure 2:**
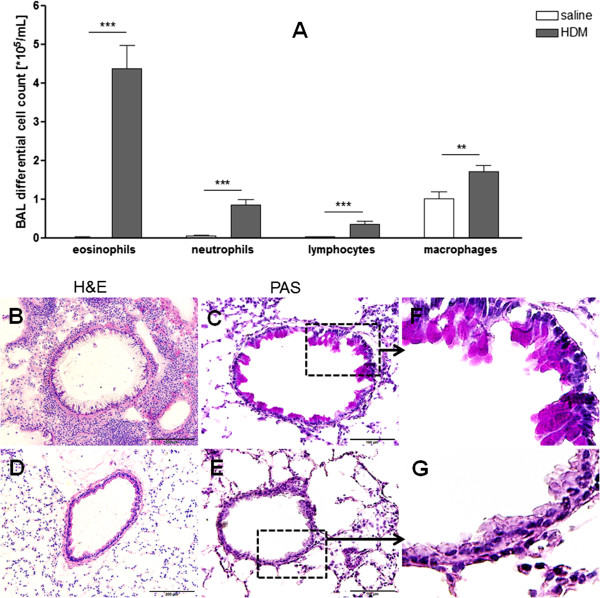
**The inflammatory response in the lungs observed after continuous expose to HDM.** Bronchoalveolar lavage fluid (BALF) cells differentiation **(A)**. Eosinophils, neutrophils, lymphocytes and macrophages in BALF demonstrated inflammation in the lungs of HDM-exposed mice, in comparison with control mice. Results are expressed as mean ± SEM. **P < 0.01, ***P < 0.001 (unpaired two-tailed t-test). Representative photomicrographs of H&E (B,D,G) and PAS (C,E,F) stained lung sections. The lung sections from HDM-sensitised and challenged mice showed an infiltration of mononuclear cells **(B)** and a highly mucus secretion **(C)** in the airways, whereas the lungs of the control mice do not contain aggregated inflammatory cells **(D)** and mucus secretion **(E). F** and **G**: larger magnifications of **C** and **E**. Scale bars of **B** and **D**: 200 μm, scale bars of **C** and **E**: 100 μm.

HDM induced chronic allergic airway inflammation is indicated by the recruitment of eosinophils, neutrophils, lymphocytes and macrophages to the lung (Figure 2A). The histological analysis with H&E and PAS staining of the lung tissues showed massive infiltration of mononuclear cells (Figure 2B) and a high mucus secretion (Figure 2C) in HDM-sensitised and -challenged mice. In contrast, the lung sections of control mice did not contain inflammatory cell aggregates (Figure 2D) and no mucus secretion was detected (Figure 2E).

### DCs are located in JNC and increased during allergic airway inflammation

**Figure 3:**
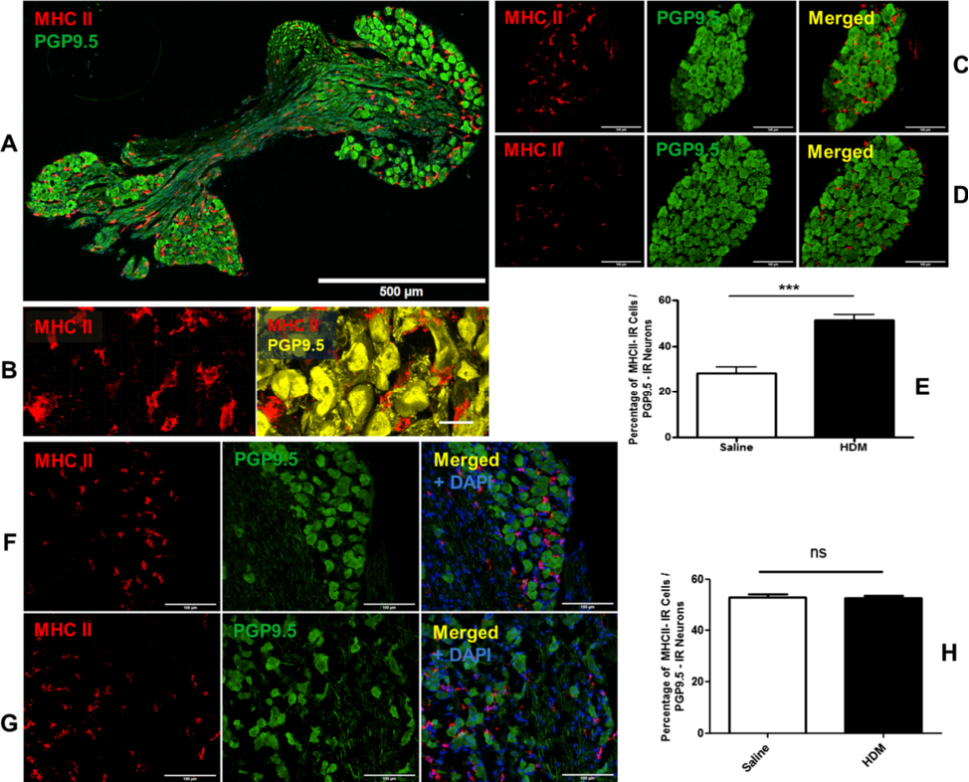
**Quantification of dendritic cells in JNC and trigeminal ganglia.** Zamboni fixed sections of JNC were stained with MHC II for DCs and PGP 9.5 for neurons. **A**: Overview of a JNC section stained for MHC II and PGP 9.5. **B**: An enlargement of DCs and neurons by using confocal microscope LSM 510 META (Carl Zeiss, Jena, Germany) with a 40x objective. Immunofluorescence analysis revealed amount of DCs, which are located next to the neuron in JNC of HDM treated mice **(C)** as well as control mice **(D)** and their number increased significantly during allergic airway inflammation. **E**: The quantification of DCs in JNC of control and HDM-treated mice. The results were expressed as percentages of MHC II positive cells in relation to PGP 9.5 positive neurons, H HDM 51.38 ± 2.38% (n = 9) vs. control 28.16 ± 2.86 (n = 10), p < 0.001. MHC II-IR cells have been found in trigeminal ganglia of control **(F)** and HDM treated mice **(G)**. **H**: The quantification of MHC II-IR cells in trigeminal ganglia of control and HDM groups. The results were expressed as percentages of MHC II-IR cells in relation to PGP 9.5 positive neurons, HDM 52.69 ± 0.86% (n = 4) vs control 52.90 ± 1.36% (n = 4). Images **A**, **C**, **D**, **F**, **G** were acquired with Olympus BX51 (Tokyo, Japan), Scale bar in **A**: 500 μm, in **B**: 20 μm, in **C**, **D**, **F**, **G**: 100 μm.

JNC sections were analysed by immunofluorescent staining for the expression of pan-neuronal marker PGP 9.5 and MHC II to identify sensory neurons and DCs and assess the anatomical localisation. Within the ganglia, neurons and nerve fibres were strongly immunoreactive for PGP 9.5 indicating that these cells were neurons and nerve fibres. Nerve fibres formed bundles crossing the ganglia. Under physiological and allergic conditions, DCs were observed to be widely distributed over the whole JNC and were located between the neurons and nerve fibres which were immunoreactive for PGP 9.5 (Figure 3).To determine how the number of DCs in JNC changed during allergic airway inflammation, DCs and neurons in JNC of HDM-treated and control mice were quantified by using MHC II- and the pan-neuronal marker PGP 9.5- immunoreactivity. The percentage of DCs in relation to the overall neurons was significantly increased in HDM-treated mice (Figure 3C) if compared to the controls (Figure 3D) (DCs/neurons: HDM 51.38 ± 2.38% (n = 9) vs. control 28.16 ± 2.86 (n = 10), p < 0.001, Figure 3E).To confirm whether the increase of DCs in JNC is caused by the targeted infiltration or global infiltration, the presence of MHC II-IR cells in trigeminal ganglia, which partly innervate the upper airways, has also been examined by immunohistochemistry. MHC II-IR cells have been found in trigeminal ganglia under physiological condition, and their numbers were not significantly changed by allergic airway inflammation (Figure 3F, G, H).

### Phenotypic characterisation of DCs in JNC

**Figure 4:**
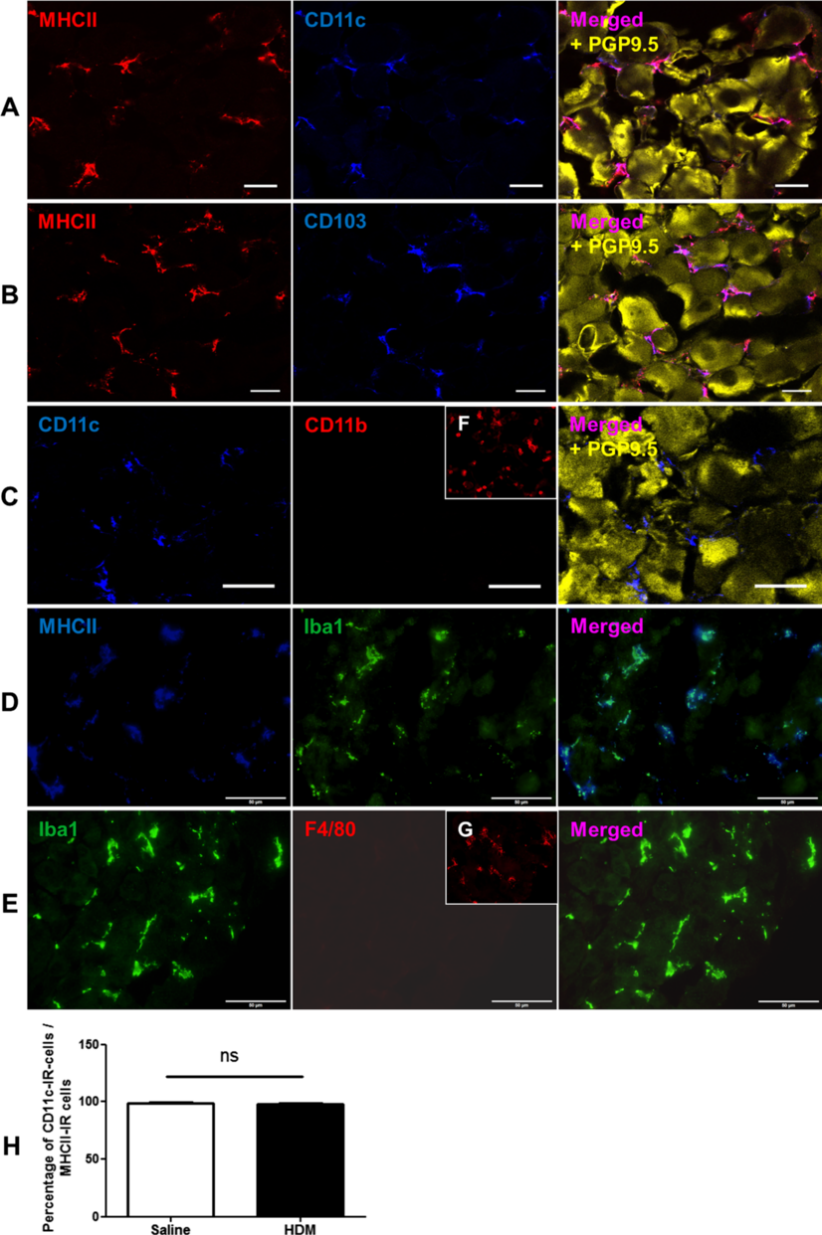
**Phenotypic characterisation of DCs in JNC.** JNC sections were stained for different cell markers and images were acquired with confocal microscope LSM 510 META (Carl Zeiss, Jena, Germany) using a 40x objective and Olympus BX51 (Tokyo, Japan) using a 20x, 40x objectives. The Immunofluorescence analysis showed the immune cells in JNC positive for MHC II and CD11c **(A)**, positive for MHC II and CD103 **(B)**, positive for CD11c and negative for CD11b **(C)**, positive for MHC II and Iba1 **(D)**, positive for Iba1 and negative for F4/80 **(E)**. **F**: Positive control of CD11b antibody in the lung tissue. **G**: Positive control of CD11c antibody in trigeminal ganglion. Yellow cells in **A**, **B**, **C** are neurons, which are stained with antibody against PGP 9.5. **H**: The quantification of CD11c-IR cells in JNC, the results were expressed as percentages of CD11c-IR cells in relation to MHC II–IR cells. Scale bars represent in **A**, **B**, **C**: 20 μm, in **D**, **E**: 50 μm.

To identify and characterise DCs phenotypically, various surface markers of DCs have been used for this study. In this respect, JNC sections were stained with antibodies against different markers of DCs such as MHC II, CD11c, CD11b, CD103, and Iba1. **To discriminate DCs from macrophages, that also express these markers, the ganglia sections were additionally stained with the antibody against F4/80, which is the best known and extensively referenced marker for mouse macrophages.** The staining results demonstrated a substantial number of cells in JNC that were strongly immunoreactive for MHC II, CD103 and Iba1. MHC II IR-cells have been found to be totally colocalised with CD103 and Iba1. The staining for CD11b and F4/80 marker did not show any immunoreactivity in MHC II IR-cells. Most of the MHC II-IR cells were immunoreactive for CD11c (CD11c-IR cells/MHC II-IR cells: HDM group: 98.84 ± 0.70% (n = 4), control group: 97.36 ± 0.60% (n = 5)). These findings demonstrated a subpopulation of DCs in JNC with these markers MHC II^+^ CD11c^+^ CD103^+^ Iba1^+^ F4/80^−^ CD11b^−^ (Figure 4).

### Discrimination between DCs and glia cells in JNC

**Figure 5:**
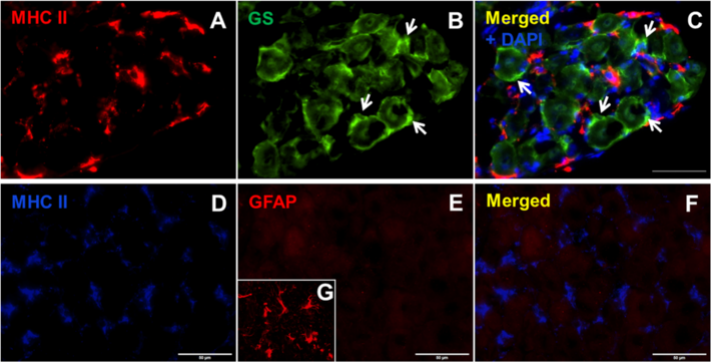
**Distinction between DCs and glia cells in JNC. A**: DCs are immunoreactive with MHC II. **B**: Satellite glia cells (SGCs, arrows) are immunoreactive for glutamine synthetase (GS). **C**: MHC II-IR cells have no overlaps with SGCs immunoreactive for GS, the separate cell nucleus of SGCs and DCs are stained with DAPI. The dendritic cells with immunoreactivity for MHC II **(D)** were also stained with astrocyte marker GFAP, these dendritic cells expressed no GFAP **(E)**. **F**: merged representation of MHC II and GFAP. **G**: positive control of GFAP antibody in mouse brain section. Scale bars: 50 μm.

The expression of MHC II molecules has been described not only in conventional antigen-presenting cells (APC) but also in human trigeminal satellite glia cells (SGCs) [29]. Therefore, the discriminations of DCs from SGCs and other glia cells in mouse airway ganglia were determined. JNC sections were stained with the SGC marker glutamine synthetase (GS), astrocyte marker glial fibrillary acidic protein (GFAP) and the APC marker MHC II. The results showed that the GS positive cells were located around neurons and formed an envelope around the sensory neurons. SGCs in JNC were found to be small-sized cells with a thin cytoplasm and a small cell nucleus without APC phenotype. These cells were negative for MHC II-immunoreactivity indicating that they were not identical with DCs, characterised with MHC II. Furthermore, GS positive cells display a different anatomical structure than the MHC II positive cells identified as DCs in JNC. Further analysis demonstrated that MHC II-IR cells in JNC are negative for GFAP and therefore they do not belong to astrocyte subpopulation of glia cells (Figure 5).

### Increase of CGRP-immunoreactive-neurons in JNC in allergic airway inflammation

**Figure 6:**
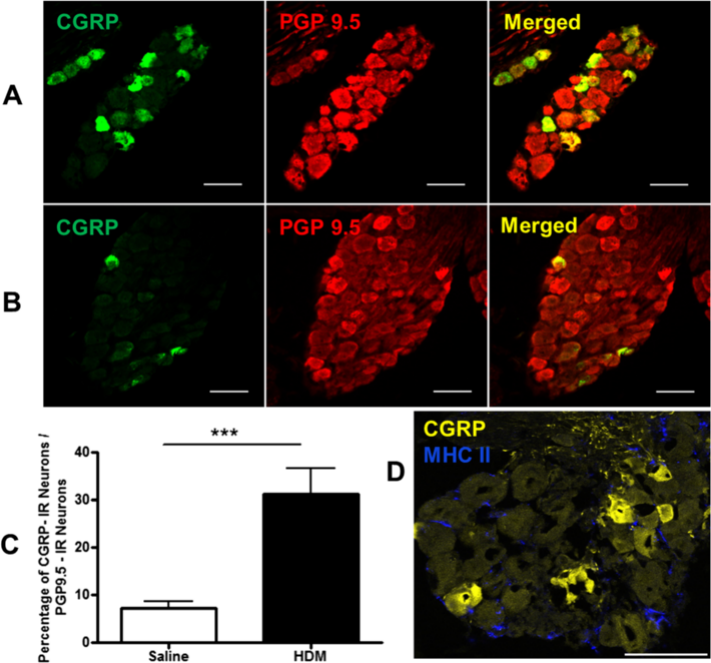
**Increased numbers of CGRP-positive neurons under allergic condition.** The JNC sections of HDM treated animals and control animals were stained with antibodies against neuropeptides CGRP and PGP 9.5. The amount of CGRP positive neurons increased significantly during allergic airway inflammation **(A)** in comparison to that of the controls **(B)**. Scale bars: 50 μm. Images were acquired with Axioskop 2 plus (Zeiss) using a 40x objective. **C**: The quantification of CGRP ir-neurons in JNC of control and HDM-treated mice. The results were expressed as percentage of CGRP-IR-neurons in relation to all PGP 9.5 positive neurons, HDM 31.16 ± 5.41% (n = 9) vs. control 7.16 ± 1.53% (n = 10), p < 0.001. **D**: MHC II-positive cells distribute overall on the JNC slices without any preferentially location to CGRP-positive neurons. Image was acquired with confocal microscope LSM 510 META (Carl Zeiss, Jena, Germany), scale bar: 50 μm.

Similarly to the finding for DCs, the percentage of CGRP ir-neurons in relation to the JNC-overall neurons was also found to be significantly elevated in HDM-treated mice (Figure 6A) when compared to the control mice (Figure 6B) (CGRP-positive neurons/total neurons: HDM 31.16 ± 5.41% (n = 9) vs. control 7.16 ± 1.53% (n = 10), p < 0.001, Figure 6C). Additionally, MHC II-positive cells have been found to have an overall distribution on the JNC slices without any preferentially location to CGRP-positive neurons (Figure 6D).

### Proliferating DCs in JNC during allergic airway inflammation

**Figure 7:**
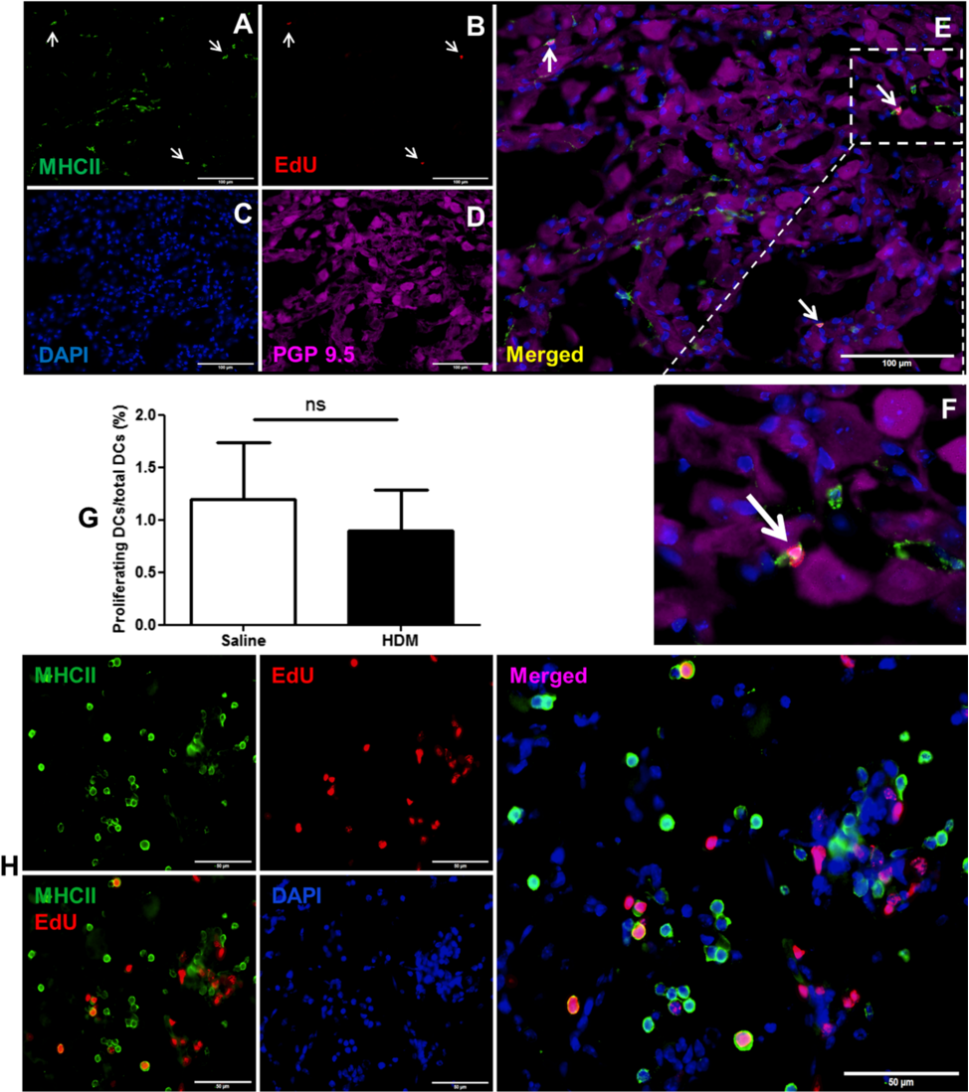
**Quantitative analysis of proliferating DCs in JNC. A**: MHC II staining of DCs. **B**: EdU detection of proliferating DCs (arrows). **C**: Nucleus staining with DAPI. **D**: Neurons positive for PGP 9.5. **E**: Merged representation of the images shown in **A-D**. **F**: An enlargement of EdU positive cell. **G**: Quantification of proliferating DCs in JNC of HDM treated and control mice. Ganglion sections were stained for EdU, MHC II, PGP 9.5 and nucleus. The proliferating DCs, which are positive with MHC II and EdU labelled nucleus, and total number of DCs were evaluated. The results were expressed as percentages of proliferating DCs/total DCs. The proliferation rate of DCs in HDM treated mice 0.89 ± 0.38% (n = 4) and control mice 1.19 ± 0.54% (n = 5), p = 0.68. **H**: The positive control of EdU in the lung. The figures showed amount of proliferating MHC II-IR cells and other cell types in the lung. Scale bars: 100 μm in **A**, **B**, **C**, **D**, 50 μm in **H**.

To examine whether DCs proliferated in JNC or migrated from outside into the ganglia during allergic airway inflammation, the animals were injected with EdU, which is incorporated with DNA of dividing cells. Systemic administration of EdU 24 h before analysis assured the detection of proliferating cells. The proliferating DCs in two groups were evaluated and expressed as percentage of MHC II positive cells with EdU-labelled nucleus/total MHC II positive cells. The results showed no significant change of proliferating DCs ratio between two groups (proliferating DCs/ total DCs: HDM 0.89 ± 0.38%, (n = 4) vs. control 1.19 ± 0.54%, (n = 5), p = 0.68, Figure 7G). Additionally, proliferation of neurons as well as SGCs was not found in the experiments. Further EdU experiments for the positive controls have been carried out in lung tissue of HDM treated mice. The finding showed a large population of MHC II-IR cells of the lung also strongly positive for EdU, showing the DCs proliferation in the lung (Figure 7H).

## Discussion

Dendritic cells play a pivotal role as antigen sampling and presenting cells in the initiation and development of allergic response such as allergic airway inflammation [3,4,8]. Previous studies have revealed that DCs were found to be localised in the neighbourhood of peripheral CGRP-positive nerve fibres in the airways [22]. The role of this DC-nerve interaction in allergic airway inflammation, however, has not been clear yet.

CGRP has been discussed to have the capacity to modulate DCs during the allergic airway inflammation. CGRP is known to be produced by the cell bodies located in JNC, anterogradely transported and released by the peripheral nerve fibres innervating the airways [12-14]. In this respect, the present study aimed to investigate the localisation, distribution and proliferation of DCs in JNC by using HDM mouse model for allergic airway inflammation.

After continuous intranasal administration with HDM extract, a chronic airway inflammation was induced as previously reported [27]. In accordance to other studies, inhalation of HDM extract led to a significant increase of the recruitment of eosinophils as well as a substantial number of neutrophils, macrophages and lymphocytes [27,28]. With the characteristic of mixed **eosinophilic** and neutrophilic inflammation, the HDM mouse model becomes an interesting animal model that may closely reflect the situation of severe bronchial asthma in human [1].

Interestingly, for the first time, cells expressing MHC II molecules were identified in JNC of mice under physiological conditions. The immunofluorescence analysis revealed that these cells belong to the immune cell population rather than to the nervous system as they were non-reactive for the pan-neuronal marker PGP 9.5. Additionally, these immune cells exhibited DC phenotypes as they displayed high immunoreactivity for MHC II and CD103 while negative for CD11b- and F4/80-immunoreactivity. Most (about 98%) of MHC II-IR cells were immunoreactive for CD11c indicating these cells are DCs. MHC II-IR cells have also been found to be totally colocalised with Iba1, which has been reported to be expressed in DCs in the brain and other organs such as intestine and skin [5,30,31]. A very small population with MHC II-IR did not express CD11c (<2%). These cell types are still unknown and remain to be investigated in future study.

The localisation of DCs within the sensory ganglia was found to be widely distributed on the whole ganglia and between SGCs and/or neurons. Recently, other neuronal cells, such as SGCs in human trigeminal ganglion, have also been shown to express MHC II [29]. However, the present findings revealed that SGCs in mouse JNC to be negative for MHC II. The contrary findings of the SGC population may be caused by the ganglionic- and species-specific differences between mouse and human [29,32]. The exact role of SGCs in JNC under physiological and allergic situations remains to be investigated in future experiments.

For the discrimination of DC migration into the ganglia as a targeted infiltration and systemic infiltration, further studies have been carried out in trigeminal ganglia. In contrast to the JNC, where the numbers of DCs were significantly increased during allergic airway inflammation, the numbers of MHC II-IR cells in trigeminal ganglia, however, were not changed. These contrary findings may be caused by the ganglionic- or organ-specific differences between JNC and trigeminal ganglia. Anatomically, trigeminal ganglia are located inside the cranium, whereas JNC are found to be under the skullbase. Additionally, the two ganglia have different embryonic development.

The findings of the increase of DCs in the JNC lead to further questions of their origin. The results of the proliferation study suggest that DCs may move from the systemic blood circulation into JNC by passing through the ganglion-blood barrier. In contrast to the brain–blood barrier, the ganglion-blood barrier is known to be only discontinuously formed [33]. Alternatively, DCs may reach the JNC along the vagus from the peripheral airways by retrograde migration when their numbers in the airways and the blood system were to be enhanced during allergic airway inflammation [34,35]. The presence of DCs in JNC also may play a substantial role in the immune response to protect the neurons against viral and bacterial infection, virus replication and bacterial spread without any neuronal destruction [36-39]. In this respect, other immune cells like T cells and macrophages were recently found inside the HSV-1 latently infected trigeminal ganglia [36]. However, the origin and function of the DCs in JNC under normal and allergic airway conditions remains to be answered in future studies.

With respect to the neuroimmune interaction, maturation, migration and function of DCs during allergic airway inflammation have been reported to be modulated by neuropeptides like the calcitonin gene-related peptide (CGRP), tachykinins and vasoactive intestinal peptide (VIP) released from airway nerves fibers [9,10,13,14]. In view of modulatory effects of CGRP on DCs, sensory neurons in JNC were investigated for CGRP-expression. The significant increase of CGRP-IR-neurons and the elevated numbers of DCs in allergic airway response suggest that there may be a functional relationship between DCs and CGRP-IR-neurons.

The increased of CGRP-IR-neurons in HDM-treated mice may have an influence on the migration, proliferation or function of DCs in JNC. With respect to the effect of CGRP in allergic airway inflammation, a proinflammatory or antiinflammatory role of CGRP in allergic airway inflammation is therefore still controversially discussed [9,10,12,40]. The intragangionic release of CGRP within JNC has not been demonstrated so far in vivo. Previous study on rat has reported about the release of CGRP from isolated JNC after stimulation with different substances such as capsaicin, nitric oxide donor sodium nitroprusside [41]. Our findings showed an increase of CGRP-positive neurons in JNC, but a release of CGRP from the neuron into the ganglia could not be demonstrated in the present study. Based on this observation, we suggest that CGRP may be paracrinely released from the neurons or anterogradely transported to the peripheral nerve endings and liberated into the airways. The exact role of CGRP in sensory neurons concerning the modulation of DC-function during allergic airway response remains to be elucidated in future studies.

## Conclusion

The present study revealed for the first time the existence of immune cells with DC phenotype within JNC. The increase of DCs and CGRP-positive neurons in airway ganglia caused by allergic airway inflammation indicates that ganglia DCs and neurons expressing CGRP may contribute to the pathogenesis of bronchial asthma. These findings demonstrate that DCs may migrate from outside into the ganglia to interact with sensory neurons enhancing or protecting the allergic airway inflammation. Further studies should be carried out to explore the interaction of DCs and neurons in airway sensory ganglia during allergic airway inflammation.

## Abbreviations

APC: Antigen-presenting cell; APES: 3-Aminopropyltriethoxysilane; BALF: Bronchoalveolar lavage fluid; CGRP: Calcitonin gene-related peptide; DC: Dendritic cells; DAPI: 4’, 6-diamidino-2-phenylindol dihydrochloride; EdU: 5-ethynyl-2’-deoxyuridine; GFAP: Glial fibrillary acidic protein; GS: Glutamine synthetase; HDM: House dust mite; H&E: Hematoxylin and eosin; Iba1: Ionized calcium binding adapter molecule 1; IR: Immunoreactive; JNC: Jugular-nodose ganglia complex; LPS: Lipopolysaccharide; MHC II: Major histocompatibility class II; PAS: Periodic Acid Schiff; PBS: Phosphate buffered saline; PGP 9.5: Protein gene product 9.5; SGC: Satellite glia cell; VIP: Vasoactive intestinal peptide.

## Competing interests

None of the authors have a financial relationship with a commercial entity that has an interest in the subject of this manuscript.

## Authors’ contributions

The authors AF, TT, RB, TW, AB, MS QTD designed the study. DDL, SR, SH performed animal experiments and analysed the data. DDL, QTD wrote the manuscript. All authors read and approved the manuscript.
